# Estimated Number of Symptomatic Lyme Borreliosis Cases in Adults in Finland in 2021 Using Seroprevalence Data to Adjust the Number of Surveillance-Reported Cases: A General Framework for Accounting for Underascertainment by Public Health Surveillance

**DOI:** 10.1089/vbz.2022.0051

**Published:** 2023-04-12

**Authors:** Julia Olsen, Frederick J. Angulo, Andreas Pilz, Kate Halsby, Patrick Kelly, Juha Turunen, Heidi Åhman, James Stark, Luis Jodar

**Affiliations:** ^1^ Vaccines Medical Development & Scientific/Clinical Affairs, Pfizer, Inc., Collegeville, Pennsylvania, USA.; ^2^ Vaccines, Pfizer Corporation Austria, Vienna, Austria.; ^3^ Vaccines, Pfizer Oy Finland, Helsinki, Finland.

**Keywords:** Lyme borreliosis, tick-borne disease, surveillance, seroprevalence, incidence, Finland

## Abstract

**Background:**

Finland conducts public health surveillance for Lyme borreliosis (LB) based on clinically diagnosed and laboratory-confirmed cases. We used data from seroprevalence studies to determine the extent to which LB cases were underascertained by public health surveillance.

**Methods:**

The numbers of incident symptomatic LB cases in 2011 in six regions in Finland were estimated using (1) data from *Borrelia burgdorferi* sensu lato seroprevalence studies, (2) estimates of the proportion of LB infections that are asymptomatic, and (3) estimates of the duration of LB antibody detection. The numbers of estimated incident symptomatic LB cases were compared with the numbers of surveillance-reported LB cases to estimate regional underascertainment multipliers. Underascertainment multipliers were applied to the numbers of surveillance-reported LB cases in each region in 2021 and summed to estimate the number of symptomatic LB cases in Finland among adults in 2021. A sensitivity analysis evaluated the impact of different durations of antibody detection.

**Results:**

Using an asymptomatic proportion of 50% and a 10-year duration of antibody detection, the estimated regional underascertainment multipliers in Finland ranged from 1.0 to 12.2. Applying the regional underascertainment multipliers to surveillance-reported LB cases in each region and summing nationally, there were 19,653 symptomatic LB cases in Finland among adults in 2021 (526/100,000 per year). With 7,346 surveillance-reported LB cases in Finland among adults in 2021, the estimated number of symptomatic LB cases indicate that there were 2.7 symptomatic LB cases for every surveillance-reported LB case among adults. With a 5- or 20-year duration of antibody detection, there were an estimated 36,824 or 11,609 symptomatic LB cases among adults in 2021, respectively.

**Discussion:**

Finland has robust public health surveillance for LB, but cases are underascertained. This framework for estimating LB underascertainment can be used in other countries that conduct LB surveillance and have conducted representative LB seroprevalence studies.

## Introduction

Lyme borreliosis (LB), the most common tick-borne disease in Europe, is caused by the spirochete *Borrelia burgdorferi* sensu lato (s.l.) (Cardenas-de la Garza et al, 2019). Clinical manifestations of LB include erythema migrans (EM) and several forms of disseminated LB (*e.g.*, neuroborreliosis and Lyme arthritis) (Cardenas-de la Garza et al, 2019).

Public health surveillance for LB has been conducted in Finland through mandatory reporting of laboratory-confirmed cases by clinical laboratories since 1995 and mandatory reporting of clinically diagnosed cases by clinicians since 2011 (Feuth et al, 2020; Finnish Institute for Health and Welfare [THL], 2022c; Sajanti et al, 2017). Laboratory-confirmed LB cases are identified by clinical laboratories through a two-tier testing approach of a sensitive enzyme immunoassay followed by a more specific immunoblot to detect borrelia-specific Immunoglobulin M/Immunoglobulin G (IgG) antibodies. Clinical laboratories report laboratory-confirmed LB cases, which are predominantly disseminated LB cases, to the National Infectious Disease Register (NIDR) (Feuth et al, 2020). Clinicians report clinically diagnosed LB cases, which are predominantly EM cases but may also include disseminated LB cases, to the National Register of Primary Health Care Visits (Avohilmo) (Feuth et al, 2020).

Although Finland has a comprehensive public health surveillance program that captures both laboratory-confirmed disseminated LB cases reported through NIDR, and clinically diagnosed EM and disseminated LB cases reported through Avohilmo, public health surveillance may fail to ascertain symptomatic LB cases for a number of reasons, including (1) persons with symptomatic LB may not seek medical care, (2) persons with symptomatic LB may seek medical care but clinicians may not diagnose LB, (3) persons with symptomatic LB may seek medical care but diagnostic specimens may not be collected, (4) diagnostic specimens may be collected from persons with symptomatic LB but appropriate tests for LB may not be performed, (5) clinicians may not report clinically diagnosed or laboratory-diagnosed LB cases, or (6) laboratories may not report laboratory-confirmed LB cases.

In addition, Avohilmo does not yet cover occupational and private health care visits, and thus likely underestimates the number of clinically diagnosed cases, especially among working-age persons.

Underascertainment of symptomatic cases occurs with almost all infectious diseases in all public health surveillance systems (Gibbons et al, 2014). Previous studies conducted in other countries and for other diseases have estimated the extent of underascertainment (*i.e.*, estimated the underascertainment “multipliers”), and then applied the estimated underascertainment multipliers to the surveillance-reported cases to estimate the total number of cases in a population. One approach for estimating underascertainment multipliers, which has been applied to a variety of infectious diseases, is to compare the number of estimated symptomatic infections derived from seroprevalence studies to the number of surveillance-reported cases (Angulo et al, 2021; Mead et al, 1999; Reed et al, 2009; Scallan et al, 2011a,; Scallan et al, 2011b; Stoner et al, 2022). This approach to estimate underascertainment multipliers for LB has not previously been reported in the literature.

We sought to estimate the total number of symptomatic LB cases among adults in Finland after adjusting the number of surveillance-reported LB cases for underascertainment.

## Materials and Methods

### Seroprevalence data

Seroprevalence studies for LB in Finland were identified through two methods: (1) a systematic literature review of PubMed, EMBASE, and CABI Direct (Global Health) from 2005 to 2020, described elsewhere (Burn et al, 2023) and (2) an additional PubMed literature search from 1997 to 2004. Identified studies were evaluated for bias using the Joanna Briggs Institute Critical Appraisal Checklist for Studies Reporting Prevalence Data (Munn et al, 2015). No ethics approval was needed because this study involved a review of published summary data with no means of identifying individual patients.

### Surveillance data

Public health surveillance data were retrieved from the Finnish Institute for Health and Welfare website (Finnish Institute for Health and Welfare [THL], 2022a; Finnish Institute for Health and Welfare [THL], 2022b). The number of reported cases from public health surveillance data conducted in the same regions as the seroprevalence studies for the same time period and among the same age range as the more recent seroprevalence study was extracted. A study by Feuth et al (2020) found a 6.3% overlap between the NIDR and Avohilmo surveillance systems, a reasonable amount of overlap since laboratory-confirmed cases could be reported in both systems. Therefore, when estimating the total number of surveillance-reported LB cases, we summed the number of microbiologically confirmed LB cases from NIDR and clinically diagnosed LB cases from Avohilmo and adjusted for the 6.3% overlap.

### Additional literature searches

Three additional literature searches were conducted to identify data inputs needed to estimate underascertainment multipliers. One, a PubMed search was conducted from 1997 to 2021 to identify the proportion of persons in Europe infected with *B. burgdorferi* s.l. who are asymptomatic. A second PubMed literature search from 1990 to 2021 was conducted to derive an estimate of the duration of antibody detection in persons infected with *B. burgdorferi* s.l. in Europe. Finally, we also conducted a PubMed literature search from 1990 to 2021 to determine the *B. burgdorferi* genospecies distribution in Finland.

### Estimation of the number of symptomatic LB cases

The number of surveillance-reported LB cases was adjusted for underascertainment using seroprevalence-derived multipliers in five steps. First, estimates of the prevalence of LB infection in the general population were identified from seroprevalence studies reporting the prevalence of *B. burgdorferi* s.l. antibodies in blood samples collected from the general population. Next, an estimate of the number of incident symptomatic LB cases was derived from the prevalence of LB infections, taking into account the proportion of infections that are asymptomatic and the duration of antibody detection in the seroprevalence studies.

Then, the number of reported cases obtained from national public health surveillance as described earlier was compared with the estimated number of symptomatic incident cases to estimate the underascertainment multiplier. Finally, the underascertainment multiplier was used to adjust the number of surveillance-reported cases from the most recent year to estimate the number of symptomatic LB cases among adults in the most recent year.

Estimates of the prevalence of LB-infected persons derived from the identified seroprevalence studies were used to estimate the number of incident symptomatic cases that occurred among adults in each region in the last year that the blood samples were collected in the more recent seroprevalence study using the formula as follows:

$$EstimatednumberofincidentsymptomaticLBcases=\frac{Seroprevalence\times populationsize\times 50\%asymptomatic}{10yeardurationofantibodydetection}.$$

The proportion of infected persons who are asymptomatic was estimated from three studies, for which the median proportion of infected persons who were asymptomatic was 50% (Hofhuis et al, 2013; Markowicz et al, 2021; Wilhelmsson et al, 2016). Our literature search for the duration of antibody detection yielded five studies, of which the median duration of IgG antibody persistence was 10 years (Glatz et al, 2006; Hammers-Berggren et al, 1994; Kalish et al, 2001; Peltomaa et al, 2003; Woudenberg et al, 2020). There was no apparent difference in the duration of antibody detection for EM versus disseminated LB cases. There is a diversity of *B. burgdorferi* genospecies in *Ixodes ricinus* ticks that cause LB (Sormunen et al, 2016).

Since the proportion of infections that are asymptomatic and the duration of antibody detection might vary by *B. burgdorferi* genospecies, we compared the genospecies distribution in Finland with the genospecies distribution in the articles used to estimate the proportion of infections that are asymptomatic and duration of antibody detection (Junttila et al, 1999; Laaksonen et al, 2018; Wilhelmsson et al, 2013). The literature search revealed that the genospecies distribution in Finland is similar to the genospecies distribution in the articles used to estimate the proportion of infections that are asymptomatic (Supplementary Table S1). The studies used to estimate the duration of antibody detection were conducted in countries (Germany, Austria, and Sweden) that are generally thought to have similar genospecies distributions to that of Finland.

The number of incident symptomatic cases among adults in each region in the last year that blood samples were collected for the more recent seroprevalence study was estimated using the formula presented earlier, and these regional estimates were summed to estimate the national number of incident symptomatic LB cases among adults.

The estimated number of incident symptomatic cases among adults in each region in the past year that blood samples were collected for the more recent seroprevalence study was also compared with the number of surveillance-reported cases among adults in each region in the same year to derive estimates of regional underascertainment multipliers.

The regional underascertainment multipliers were then applied to the number of surveillance-reported cases among adults in each region in 2021 and summed to estimate the total number of symptomatic LB cases in Finland in 2021. To derive a national underascertainment multiplier, the total estimated number of symptomatic LB cases among adults in Finland in 2021 was compared with the total number of surveillance-reported cases among adults in Finland in 2021.

### Sensitivity analyses

We conducted a sensitivity analysis to evaluate the impact of using 5- and 20-year durations of antibody detection. In addition, underascertainment multipliers were derived using only NIDR (laboratory-confirmed surveillance-reported cases) and Avohilmo (clinician-diagnosed surveillance-reported cases) to estimate the extent of underascertainment if Finland only had one of these two surveillance systems. A third sensitivity analysis was performed to estimate the underascertainment multipliers and the estimated number of symptomatic LB cases in 2021 among the entire population of Finland, under the assumption that the seroprevalence reported in the seroprevalence study is representative for all ages.

## Results

**FIG. 1.**
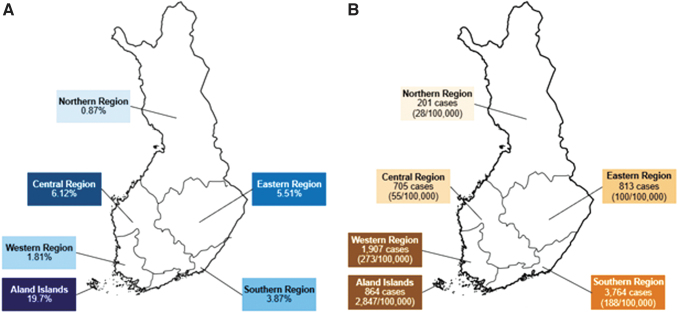
**(A)** Seroprevalence of IgG antibodies against *Borrelia burgdorferi* from a 2011 national seroprevalence study (van Beek et al., 2018) and a 1993–1997 study on Åland Islands (Carlsson et al., 1998), by region, Finland. **(B)** Total number of reported LB cases (population-based incidence of reported LB cases) [Finnish Institute for Health and Welfare (THL), 2022a; Finnish Institute for Health and Welfare (THL), 2022b], adjusting for the overlap between the surveillance systems, Finland, 2021.

A nationally representative seroprevalence study, with blood samples collected from adults (29–97 years) in 2011 during a national health survey (van Beek et al, 2018), reported the seroprevalence of detectable IgG antibodies against *B. burgdorferi* s.l. in five regions of Finland (Northern, Eastern, Central, Western, and Southern) (Fig. 1). As the national health survey did not include the Åland Islands, an earlier seroprevalence study (Carlsson et al, 1998), with blood samples collected between 1993 and 1997, was used to estimate the seroprevalence for the Åland Islands. The risk of bias in these two seroprevalence studies was judged to be low using the Briggs Checklist (Supplementary Table S2). Based on the seroprevalence study results, with a 50% asymptomatic proportion and 10-year duration of antibody detection, there were an estimated 7153 incident symptomatic LB cases among adults ≥30 years in 2011 (Table 1).

**Table 1.** Estimated Number of Incident Lyme Borreliosis Cases Among Adults ≥30 Years Derived from a 2011 National Seroprevalence Study (van Beek et al., 2018) and a 1993–1997 Study on Åland Islands (Carlsson et al., 1998), by Region, Finland, 2011

Region	2011 Seroprevalence (%)	2011 Population size (adults ≥30 years)	Estimated number of incident cases among adults ≥30 years in 2011
Northern	0.87	456,973	199
Eastern	5.51	559,850	1,542
Central	6.12	817,225	2,501
Western	1.81	460,329	417
Southern	3.87	1,192,895	2,308
Åland Islands	19.7	18,928	186
Nationwide	—	3,506,200	7,153

**FIG. 2.**
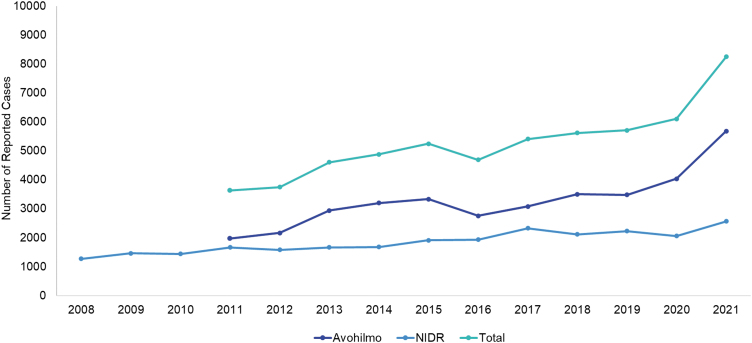
Total number of reported LB cases, adjusting for the overlap between the surveillance systems, in Finland from 2011 to 2021 [Finnish Institute for Health and Welfare (THL), 2022a; Finnish Institute for Health and Welfare (THL), 2022b].

Overall, there were 8,254 surveillance-reported LB cases in 2021 (Figs. 1B and 2), corresponding to an incidence of 153 surveillance-reported LB cases per 100,000 population in 2021 (Table 2). In 2011, the nationwide incidence was 67 per 100,000 population. Among adults ≥30 years, there were 5,490 LB cases reported to Avohilmo and 2,202 LB cases reported to NIDR in 2021. After adjusting for the overlap between the surveillance systems, there were 7,346 surveillance-reported LB cases among adults ≥30 years in 2021, for a nationwide incidence of 196 surveillance-reported LB cases per 100,000 population in 2021. The incidence of surveillance-reported LB cases in 2021 represents an increase from the incidence of 89 per 100,000 population among adults ≥30 years in 2011.

**Table 2.** Annual Surveillance-Reported Lyme Borreliosis Incidence, by Region, Finland, 1995, 2011, and 2021

Region	Annual incidence (unadjusted for underascertainment)		
	1995^a^	2011	2021
Northern	1/100,000	7/100,000	28/100,000
Eastern	4/100,000	44/100,000	100/100,000
Central	2/100,000	21/100,000	55/100,000
Western	16/100,000	88/100,000	273/100,000
Southern	7/100,000	87/100,000	188/100,000
Åland Islands	258/100,000	2,617/100,000	2,847/100,000
Nationwide	7/100,000	67/100,000	153/100,000

^a^ 1995 is the oldest year of accessible data on the Finnish Institute for Health and Welfare's website. Avohilmo reporting began in 2011; thus, only NIDR data were used for the 1995 incidence calculation. Number of cases used in calculations were adjusted for the 6.3% overlap between Avohilmo and NIDR.

Using a 50% asymptomatic proportion and a 10-year duration of antibody detection, regional underascertainment multipliers in 2011 ranged from 1.0 in the Western region and the Åland Islands to 12.2 in the Central region (Table 3). When the regional underascertainment multipliers were applied to the number of surveillance-reported LB cases (adjusted for overlap) in 2021 and then summed, there were an estimated 19,653 symptomatic LB cases nationwide among adults >30 years in Finland in 2021 (Table 3). Compared with the number of surveillance-reported LB cases among adults >30 years in 2021 (7,346), the estimated number of symptomatic LB cases indicates that the nationwide underascertainment multiplier of symptomatic LB cases is 2.7.

**Table 3.** Underascertainment Multipliers and Estimates of Symptomatic Lyme Borreliosis Cases, Adults ≥30 Years, by Region, Finland, 2021

Region	Total number of reported LB cases among adults ≥30 years in 2011	Estimated number of incident LB cases among adults ≥30 years in 2011	Multiplier (based on 2011 data)	Total number of reported LB cases among adults ≥30 years in 2021	Estimated number of symptomatic LB cases among adults ≥30 years in 2021
Northern	38	199	5.3	154	811
Eastern	319	1,542	4.8	705	3,414
Central	206	2,501	12.2	593	7,207
Western	539	417	1.0	1,703	1,703
Southern	1,372	2,308	1.7	3,411	5,739
Åland Islands	637	186	1.0	779	779
Nationwide	3,110	7,153	2.7^a^	7,346	19,653

^a^ Nationwide multiplier is derived from regional multipliers, weighted by the population size of each region; therefore, nationwide multiplier is not simply the ratio of the national estimated number of incident LB cases in 2011 to the national number of reported LB cases in 2011.

LB, Lyme borreliosis.

### Sensitivity analyses

In the sensitivity analysis, using a 5-year duration of antibody detection, the regional underascertainment multipliers ranged from 1.0 to 24.3. When these multipliers were applied to the surveillance-reported LB cases in 2021, this resulted in an estimated 36,824 symptomatic LB cases in Finland in 2021 for a nationwide underascertainment multiplier of 5.0 (Table 4). Using a 20-year duration of antibody detection, the regional underascertainment multipliers ranged from 1.0 to 6.1 and there were an estimated 11,068 symptomatic LB cases in Finland in 2021, for a nationwide underascertainment multiplier of symptomatic LB cases of 1.5.

**Table 4.** Sensitivity Analysis: Underascertainment Multipliers and Estimate of Symptomatic Lyme Borreliosis Cases, Adults ≥30 Years, by Region, Finland, 2021, Using 5- and 20-Year Durations of Antibody Detection

Region	Total number of reported LB cases among adults ≥30 years in 2011	Estimated number of incident LB cases among adults ≥30 years in 2011		Multiplier (based on 2011 data)		Total number of reported LB cases among adults ≥30 years in 2021	Estimated number of LB cases among adults ≥30 years in 2021	
		5 Years	20 Years	5 Years	20 Years		5 Years	20 Years
Northern	38	398	99	10.5	2.6	154	1,623	406
Eastern	319	3,085	771	9.7	2.4	705	6,828	1,707
Central	206	5,001	1,250	24.3	6.1	593	14,414	3,604
Western	539	833	208	1.5	1.0	1,703	1,703	1,703
Southern	1,372	4,617	1,154	3.4	1.0	3,411	11,477	3,411
Åland Islands	637	373	93	1.0	1.0	779	779	779
Nationwide	3,110	14,306	3,577	5.0^a^	1.6^a^	7,346	36,824	11,609

^a^ Nationwide multiplier is derived from regional multipliers, weighted by the population size of each region; therefore, nationwide multiplier is not simply the ratio of the national estimated number of incident LB cases in 2011 to the national number of reported LB cases in 2011.

If Finland only had the clinician-based Avohilmo LB surveillance system or only had the laboratory-based NIDR LB surveillance system, the estimated nationwide underascertainment multipliers for symptomatic LB cases would be 4.0 and 5.0, respectively (Table 5).

**Table 5.** Sensitivity Analysis: Underascertainment Multipliers of Clinician-Based Surveillance and Laboratory-Based Surveillance Only, Adults ≥30 Years, by Region, Finland

Region	Estimated number of incident cases among adults ≥30 years in 2011	Total number of reported cases among adults ≥30 years in 2011		Multiplier	
		Avohilmo	NIDR	Clinician-based (Avohilmo-only)	Lab-based (NIDR-only)
Northern	199	20	19	9.9	10.5
Eastern	1,542	260	75	5.9	20.6
Central	2,501	146	69	17.1	36.2
Western	417	314	245	1.3	1.7
Southern	2,308	822	602	2.8	3.8
Åland Islands	186	227	424	1.0	1.0
Nationwide	7,153	1,789	1,434	4.0^a^	5.0^a^

^a^ Nationwide multiplier is derived from regional multipliers, weighted by the population size of each region; therefore, nationwide multiplier is not simply the ratio of the national estimated number of incident LB cases in 2011 to the national number of reported LB cases in 2011.

NIDR, National Infectious Disease Register.

We also conducted a sensitivity analysis in which we assumed the seroprevalence study results are representative of the entire population of Finland, including children. In this analysis, the regional underascertainment multipliers in 2011 ranged from 1.0 to 14.6 (Supplementary Table S3). When the regional underascertainment multipliers were applied to the number of surveillance-reported LB cases (adjusted for overlap) in 2021 and then summed, there were an estimated 27,907 symptomatic LB cases nationwide in Finland in 2021 (Supplementary Table S3). Compared with the number of surveillance-reported LB cases in 2021 (8,254), the estimated number of symptomatic LB cases indicates that the nationwide underascertainment multiplier of symptomatic LB cases is 3.4.

## Discussion

Finland has a high incidence of surveillance-reported LB cases and a robust LB public health surveillance system, which includes mandatory reporting of both clinically diagnosed LB cases and laboratory-confirmed LB cases. Nevertheless, our seroprevalence-derived estimates of the underascertainment multipliers indicate that symptomatic LB cases are underascertained in Finland. According to our estimates, there were an estimated 2.7 symptomatic LB cases for every surveillance-reported LB case in Finland among adults ≥30 years in 2021.

The seroprevalence estimates and underascertainment multipliers in Finland vary by region. The seroprevalence estimate of 0.87% in the Northern region was the lowest regional estimate, which reflects low surveillance-reported LB incidence due to fewer tick populations in Northern Finland, especially in the Arctic Circle region (Laaksonen et al, 2017). There is a higher tick density and higher LB incidence in the more Southern regions in Finland. Reasons for the larger underascertainment multipliers in the Northern, Eastern, and Central regions are not known; further studies are needed to determine if the larger underascertainment multipliers in these regions are associated with a lower awareness of LB resulting in less diagnosis and less reporting of LB cases in the upper half of Finland as compared with the lower half of the country.

The quality of the estimates of the underascertainment multipliers derived from this seroprevalence-based approach is dependent on the quality of the seroprevalence and surveillance system data. Estimation of underascertainment of symptomatic LB cases by Finland's public health surveillance system for LB was facilitated by the availability of a published nationally representative seroprevalence study in Finland that provided credible regional estimates of the proportion of the population of adults ≥30 years who have been infected with *B. burgdorferi*.

The 2011 national seroprevalence study utilized a modified two-tier testing approach, with sera samples tested by whole-cell sonicate IgG ELISA, C6 peptide ELISA, and recomBead IgG 2.0. The seroprevalence study used for the Åland Islands was of lower quality and utilized single-tier testing. In general, single-tier testing inflates the number of false positives and thus seroprevalence studies utilizing single-tier testing may provide an inaccurately high estimate of the proportion of the population that has been infected with *B. burgdorferi.*

In this analysis, however, the estimated number of incident cases in the Åland Islands based on the seroprevalence estimate was lower than the number of surveillance-reported cases in this region; thus, the use of single-tier testing in the Åland Islands seroprevalence study did not have a substantial impact on the estimation of underascertainment multipliers. In addition to the high quality of the seroprevalence studies, the public health surveillance system for LB in Finland is of high quality and comprehensive, capturing both clinically diagnosed and laboratory-confirmed LB cases. The availability of these high-quality seroprevalence studies and surveillance systems support the credibility of the estimates of the underascertainment multipliers in our study.

The estimation of seroprevalence-derived underascertainment multipliers relies on several assumptions. Using data available from the published literature, the percentage of infected persons with an asymptomatic infection was estimated to be 50%. If a higher proportion of infected cases are asymptomatic, the estimated number of symptomatic cases would be lower and vice versa. Again, using data available from the published literature, the estimation approach used a 10-year duration of IgG antibody detection. We also conducted a sensitivity analysis to evaluate the impact of shorter or longer durations of antibody detection.

With a shorter duration of antibody detection, the estimated number of symptomatic cases and underascertainment multipliers would be higher. The studies used to derive the percentage of infected persons with an asymptomatic infection and the duration of IgG antibody detection were not conducted in Finland. Given that the genospecies distribution may vary by country and impact these factors, we also used data available from the published literature to verify that the genospecies distribution in Finland is similar to the genospecies distribution in the studies used to derive these estimates. These studies also corroborate the genospecies identified within infected Ixodid ticks throughout Finland (Laaksonen et al, 2018; Laaksonen et al, 2017).

The number of surveillance-reported LB cases in Finland was lower in 2011, when the blood samples for the national seroprevalence study were collected, than in 2021. The blood samples for the seroprevalence study on the Åland Islands were collected between 1993 and 1997. In 1997, only laboratory-confirmed LB cases were nationally notifiable in Finland and the number of surveillance-reported LB cases was also lower in 1997 than in 2021. The assumption inherent in applying the underascertainment multipliers derived in 2011 to the number of surveillance-reported LB cases in 2021 is that the factors associated with being a reported LB case in the public health surveillance system (*e.g.*, frequency at which an infected person sought care and frequency of reporting of LB cases by clinicians and laboratories) remained unchanged from 2011 to 2021, although it is not possible to determine the validity of this assumption.

As both of the seroprevalence studies were conducted in adults, we estimated adult-specific underascertainment multipliers. We did not have children-specific seroprevalence data. However, we did conduct a sensitivity analysis and applied the seroprevalence results to the entire population to estimate underascertainment multipliers and the number of symptomatic LB cases in 2021 among all ages; this analysis relies on the assumption that the seroprevalence reported in the studies used reflects the seroprevalence in the entire population in each region.

According to the reported number of LB cases in national surveillance, after adjusting for overlap of the two surveillance systems, the 2021 incidence of LB was ∼78 per 100,000 in children ages 0–19 and 167 per 100,000 in adults ages 20+. This demonstrates that in Finland, LB occurs in children, although at a lower incidence than in adults. Thus, it is possible that this sensitivity analysis, through applying the seroprevalence results to the entire population, overestimates the number of incident symptomatic cases of LB in Finland in 2021.

This is a general framework that can be applied in any country that conducts public health surveillance for LB and has seroprevalence data that are representative of the prevalence of IgG antibodies against *B. burgdorferi* in the general population. This approach of estimating seroprevalence-derived multipliers to estimate the actual number of symptomatic LB cases could be utilized in other countries that conduct public health surveillance for LB provided data are available from seroprevalence studies that are representative of the general population. Despite the comprehensive LB surveillance system in Finland, there were an estimated 2.7 symptomatic LB cases for each surveillance-reported LB case among adults ≥30 years in Finland in 2021. Disease prevention efforts, including the availability of an efficacious LB vaccine, are needed to address this important public health problem.

## Conclusions

Finland has robust LB surveillance, but symptomatic LB cases are underascertained. Using a 50% asymptomatic proportion and a 10-year duration of antibody detection, regional underascertainment multipliers for adults ranged from 1.0 to 12.2, which, when applied to surveillance-reported LB cases in each region, summed to 19,653 symptomatic LB cases among adults ≥30 years in 2021. With 7,346 surveillance-reported LB cases among adults ≥30 years in Finland in 2021, the estimated number of symptomatic LB cases indicate that there were 2.7 symptomatic LB cases for every surveillance-reported LB case among adults ≥30 years in Finland. This framework for estimating LB underascertainment can be used in other countries that conduct LB surveillance and have conducted representative LB seroprevalence studies.

## Supplementary Material

Supplemental data

Supplemental data

Supplemental data
