# Supplementary material for: Estimated Number of Symptomatic Lyme Borreliosis Cases in Adults in Finland in 2021 Using Seroprevalence Data to Adjust the Number of Surveillance-Reported Cases: A General Framework for Accounting for Underascertainment by Public Health Surveillance
Source: Vector Borne Zoonotic Dis. 2023 Apr 12;23(4):265–72. doi: 10.1089/vbz.2022.0051 (PMC10122260; doi:10.1089/vbz.2022.0051)
Supplement: Supplemental data [file Suppl_TableS1.docx]

**Table S1.** Genospecies distribution of *Ixodes ricinus* ticks in Finland and of the articles used to estimate the proportion of infections that are asymptomatic

| Finland  papers | *B. afzelii* | *B. garinii* | *B. burgdorferi ss* | *B. valaisiana* |
| --- | --- | --- | --- | --- |
| Laaksonen et al., 2018  (n=394) | 40.7% | 44.6% | 5.6% | 9.1% |
| Junttila et al., 1999  (n=142) | 71% | 27% | 4% | -- |
| Wilhelmsson et al., 2013  (n=178) | 48% | 24% | 2% | 8% |
| Asymptomatic proportion papers |  |  |  |  |
| Hofhuis et al., 2013  (n=56) | 64.3% | 19.6% | 12.5% | 7.1% |
| Markowicz et al., 2021  (n=194) | 68.0% | 16.5% | 7.7% | 7.2% |
| Wilhelmsson et al., 2016^a^ | -- | -- | -- | -- |

^a^no genospecies information was reported; however, this study utilized the same study population as Wilhelmsson et al., 2013
