# Supplementary material for: Estimated Number of Symptomatic Lyme Borreliosis Cases in Adults in Finland in 2021 Using Seroprevalence Data to Adjust the Number of Surveillance-Reported Cases: A General Framework for Accounting for Underascertainment by Public Health Surveillance
Source: Vector Borne Zoonotic Dis. 2023 Apr 12;23(4):265–72. doi: 10.1089/vbz.2022.0051 (PMC10122260; doi:10.1089/vbz.2022.0051)
Supplement: Supplemental data [file Suppl_TableS2.docx]

**Table S2.** Evaluation of national (Van Beek et al., 2018) and Åland Islands (Carlsson et al., 1998) seroprevalence studies using Briggs Scale Attributes

| Attribute | National | Åland Islands |
| --- | --- | --- |
| Was the sample frame appropriate to address the target population? | Yes | Yes |
| Were study participants sampled in an appropriate way? | Yes | Yes |
| Was the sample size adequate? | Yes | Yes |
| Were the study subjects and the setting described in detail? | Yes | Yes |
| Was the data analysis conducted with sufficient coverage of the identified sample? | Yes | Yes |
| Were valid methods used for the identification of the condition? | Yes | Satisfactory |
| Was the condition measured in a standard, reliable way for all participants? | Yes | Yes |
