# Supplementary material for: Estimated Number of Symptomatic Lyme Borreliosis Cases in Adults in Finland in 2021 Using Seroprevalence Data to Adjust the Number of Surveillance-Reported Cases: A General Framework for Accounting for Underascertainment by Public Health Surveillance
Source: Vector Borne Zoonotic Dis. 2023 Apr 12;23(4):265–72. doi: 10.1089/vbz.2022.0051 (PMC10122260; doi:10.1089/vbz.2022.0051)
Supplement: Supplemental data [file Suppl_TableS3.docx]

**Table S3**. Sensitivity analysis: Under-ascertainment multipliers and estimates of symptomatic LB cases, all ages, by region, Finland, 2021

| Region | Total number of reported LB cases in 2011 | Estimated number of incident LB cases in 2011 | Multiplier  *(based on 2011 data)* | Total number of reported LB cases in 2021 | Estimated number of symptomatic LB cases in 2021 |
| --- | --- | --- | --- | --- | --- |
| Northern | 52 | 318 | 6.1 | 201 | 1,227 |
| Eastern | 368 | 2,320 | 6.3 | 813 | 5,121 |
| Central | 260 | 3,800 | 14.6 | 705 | 10,297 |
| Western | 608 | 628 | 1.0 | 1,907 | 1,969 |
| Southern | 1,611 | 3,607 | 2.2 | 3,764 | 8,429 |
| Åland Islands | 742 | 279 | 1.0 | 864 | 864 |
| Nationwide | 3,641 | 10,952 | 3.4* | 8,254 | 27,907 |

*nationwide multiplier is derived from regional multipliers, weighted by the population size of each region; therefore, nationwide multiplier is not simply the ratio of the national estimated number of incident LB cases in 2011 to the national number of reported LB cases in 2011
